# Hemoglobin Interactions with αB Crystallin: A Direct Test of Sensitivity to Protein Instability

**DOI:** 10.1371/journal.pone.0040486

**Published:** 2012-07-18

**Authors:** Tyler J. W. Clark, Scott A. Houck, John I. Clark

**Affiliations:** 1 Department of Biological Structure, University of Washington School of Medicine, Seattle, Washington, United States of America; 2 Department of Cell & Developmental Biology, University of North Carolina, Chapel Hill, North Carolina, United States of America; 3 Department of Ophthalmology, University of Washington School of Medicine, Washington, United States of America; Griffith University, Australia

## Abstract

As a small stress response protein, human αB crystallin, detects protein destabilization that can alter structure and function to cause self assembly of fibrils or aggregates in diseases of aging. The sensitivity of αB crystallin to protein instability was evaluated using wild-type hemoglobin (HbA) and hemoglobin S (HbS), the glutamate-6-valine mutant that forms elongated, filamentous aggregates in sickling red blood cells. The progressive thermal unfolding and aggregation of HbA and HbS in solution at 37°C, 50°C and 55°C was measured as increased light scattering. UV circular dichroism (UVCD) was used to evaluate conformational changes in HbA and HbS with time at the selected temperatures. The changes in interactions between αB crystallin and HbA or HbS with temperature were analyzed using differential centrifugation and SDS PAGE at 37°C, 50°C and 55°C. After only 5 minutes at the selected temperatures, differences in the aggregation or conformation of HbA and HbS were not observed, but αB crystallin bound approximately 6% and 25% more HbS than HbA at 37°C, and 50°C respectively. The results confirmed (a) the remarkable sensitivity of αB crystallin to structural instabilities at the very earliest stages of thermal unfolding and (b) an ability to distinguish the self assembling mutant form of HbS from the wild type HbA in solution.

## Introduction

Human αB crystallin is a small heat-shock protein (sHSP) that participates in a variety of degenerative processes during aging [1]–[5]. Cellular expression of αB crystallin increases in response to physical and chemical stimuli, including temperature, pH, hypoxia, osmotic pressure, proteolysis, chemical modification, oxidation, and changing metabolite levels which are characteristic of cardiovascular disease, cataract, and neurodegeneration [6]–[9]. The protective function of αB crystallin against stress depends on multiple interactive sequences that map to the exposed surface of the molecule [1], [7]. Under conditions of disease or normal cell differentiation, αB crystallin can recognize self-assembling and/or aggregating proteins [7], [10]. Weak energies of interaction between small heat shock proteins (sHSP) and destabilized mutant T4 lysozyme suggested that sHSP are general sensors for protein instability [11], [12]. The experiments in this report tested the sensitivity of αB crystallin to thermal destabilization of mutant HbS and wild type HbA prior to significant and measurable effects on conformation.

HbA and HbS were selected as the experimental models for the study of αB crystallin sensitivity to protein destabilization because the molecular basis for HbA and HbS stability in solution is very well characterized [13], [14]. Wild type hemoglobin (HbA) is found in normal red blood cells as a soluble tetramer of two α and two β monomers that are necessary and sufficient for oxygen binding [15]. The monomers are predominantly α helix and changes in conformation can be monitored readily using far ultraviolet circular dichroism (UVCD). Sickle cell anemia is caused by the hemoglobin S (HbS) mutation, a missense mutation (E6V) in the β subunit of hemoglobin [13], [15]. The HbS mutation alters the surface interactions between the hemoglobin subunits shifting the phase diagram for hemoglobin to favor formation of long, insoluble, fibril aggregates. In vivo, the assembly and elongation of the seven double stranded HbS fibrils is responsible for the deformed sickle shaped red blood cells that can block capillaries and generate the pathological phenotypes observed in sickle cell patients [16]–[18]. While hemoglobin gene expression was reported in lens [19], [20] and in neurodegenerative diseases during aging [21], [22], a functional relationship between αB crystallin and HbA or HbS in vivo is not known. When HbA or HbS are thermally destabilized, they are capable of nucleating light scattering aggregates [23], [24]. For effective protection against aggregation, early recognition of unfolding proteins, even prior to nucleation, needs to be achieved by αB crystallin. The current study determined that the sensitivity of αB crystallin to the thermal instability of normal hemoglobin (HbA) and sickle cell hemoglobin (HbS) occurred before significant conformational modification and aggregation measured by light scattering. As expected the interactions between αB crystallin and HbA or HbS were greater at higher temperatures than lower temperatures. Despite the high degree of similarity in the sequence and 3D atomic structure of HbA and HbS, the stress protein human αB crystallin distinguished HbA from HbS under modest destabilizing conditions. These results are a direct experimental demonstration of the sensitivity of αB crystallin to small differences at the surface of normal hemoglobin (HbA) and sickle cell hemoglobin (HbS) without measureable differences in conformation.

## Methods

### Materials

Ferrous stabilized human hemoglobin A_0_ (HbA) (Sigma-Aldrich H0267-25MG) and Ferrous stabilized human hemoglobin S (HbS) (Sigma-Aldrich H0392-25MG) were purchased from the manufacturer and used without further purification. Human αB crystallin was grown from E.coli and purified using ion-exchange and size exclusion chromatography as described previously [25].

Data sets for the high resolution X-ray diffraction structures of human deoxy-hemoglobinA (PDB:4HHB) and deoxy-hemoglobin S (PDB:2HBS) were obtained from the Protein Data Bank (www.PDB.org). The solvent-exposed surface area of the β subunit of hemoglobin was calculated using PyMOL (DeLano Scientific Corp.) molecular structure analysis software.

### Thermal Stress Aggregation Assay to Measure Interactions between Unfolding Proteins

Samples of 13.5 µM HbA or HbS in the presence or absence of 13.5 µM αB crystallin were dissolved in 150 mM PBS pH7.0. αB crystallin was also observed independently, as a control. 400 µl of sample was loaded into a 10 mm glass cuvette (Starna Cells, Inc), mounted in a Pharmacia Biotech Ultrospec 3000 UV/Visible Spectrophotometer, and heated at temperatures of 37, 50 or 55°C for 75 minutes. Optical density was measured as absorbance (milli-Absorbance Units, mAU) at 700 nm every 20 seconds for each sample. The progressive change in absorbance was normalized to the highest value of light scattering recorded at 55°C and the normalized O.D.(optical density) was plotted as a function of time. A background reference of PBS at the same buffer concentration was used as the baseline and the O.D. was unchanged over the 75 minute time period. Protein unfolding and aggregation was measured as an increase in light scattering at a wavelength of 700 nm to minimize absorption by the hemoglobin heme group at 405 nm.

### Sedimentation Assay to Evaluate Interactions between Proteins (Fig. 1)

**Figure 1:**
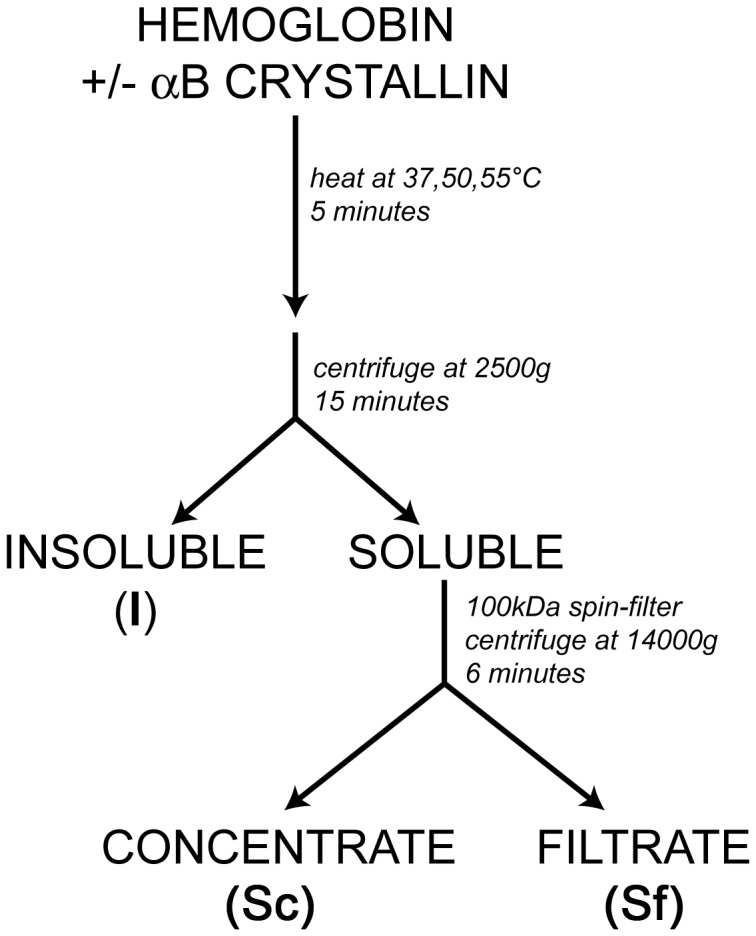
Assay for interactions between αB crystallin and HbA or HbS. Samples of Hemoglobin A or Hemoglobin S were heated in the presence and absence of αB crystallin and then the interactive aggregates or bound complexes were separated by centrifugation. At low speed (2500 g) only the largest aggregates were in the insoluble (I) fraction, and the Hb bound to αB crystallin remained soluble in the supernatant (soluble) fraction. The soluble fraction was centrifuged again at 14,000 g using a 100 kDa spin-filter to separate the HbA or HbS bound to αB crystallin in the soluble concentrate (Sc) from the soluble HbA or HbS in the filtrate (Sf). With thermal destabilization, aggregation was expected to increase the amount of αB crystallin bound HbA or HbS in the insoluble (I) and soluble concentrate (Sc) fractions and decrease the amount in the soluble filtrate (Sf) fraction. The centrifugation experiments were conducted under conditions of minimal thermal stress to determine the sensitivity of αB crystallin to the earliest stages of protein unfolding and aggregation.

A low speed centrifugation that separated soluble proteins from insoluble aggregates was followed by a high speed centrifugation of the supernatant using a 100 kDa cutoff filter to separate αB crystallin bound to HbA or HbS from unbound HbA or HbS. Samples of 13.5 µM HbA or HbS (MW = 17 kDa or 68 kDa tetramer) in the presence or absence of 13.5 µM αB crystallin (MW = 20 kD) were dissolved in 150 mM PBS pH7.0, the same conditions used in the thermal stress aggregation assays. Samples were heated at 37, 50 or 55°C for 5 minutes, then centrifuged at 2500G (6000 RPM) for 15 minutes using an Eppendorf 5415C desktop centrifuge to separate the insoluble protein aggregates from the protein which remained soluble in the supernatant (Fig. 1). To characterize interactions between HbA or HbS and αB crystallin in the soluble supernatant, a filter system was used. The supernatant was pipetted into a Microcon Filter (Ultracel YM-100 Regenerated Cellulose) with a molecular weight cutoff of 100 kDa and centrifuged at 14,000G for 6 minutes to separate HbA or HbS interacting with αB crystallin from free HbA or HbS. The filtrate was removed. The filter was then placed in another microtube and centrifuged upside-down to extract the bound proteins in the concentrate. The three samples, Insoluble (I), Soluble Concentrate (Sc), and Soluble Filtrate (Sf), were resolved by SDS-PAGE (SDS-polyacrylamide electrophoresis). The gel was scanned and densitometry was conducted on each lane using ImageJ to compare the free HbA or HbS to αB crystallin co-localized with HbA or HbS.

### Ultraviolet Circular Dichroism (UVCD) was used to Determine Structural Stability of HbA and HbS

**Figure 2:**
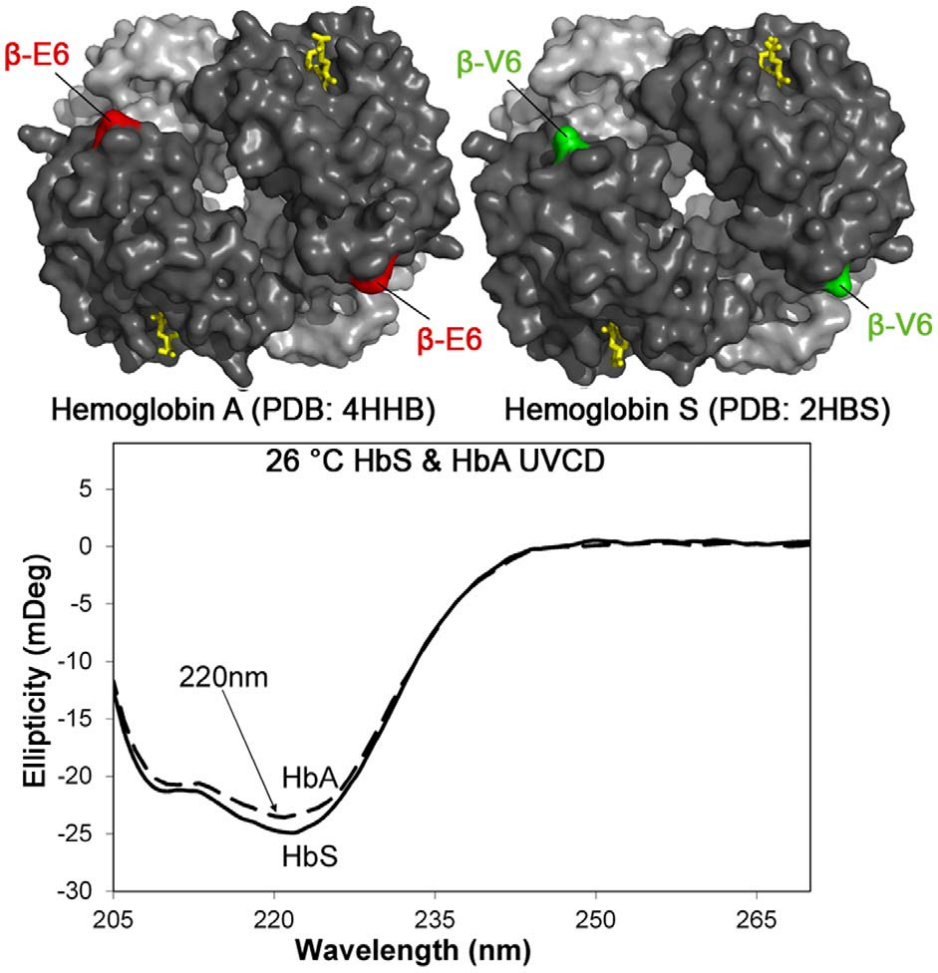
Minimal differences in the conformation of HbA and HbS. **Top Left:** The molecular structure of normal Hemoglobin A (PDB:4HHB). **Top Right:** The molecular structure of Hemoglobin S (PDB:2HBS), containing the glu6val mutation in the β chain of hemoglobin that causes sickle-cell disease. The α chain of hemoglobin is light grey. The β chain of hemoglobin is dark grey. The heme groups are yellow. The total solvent exposed surface area of the β subunit in Hb A is 23476 Å^2^ and 23246 Å^2^ in HbS which is a difference of approximately 1%. The structure of the mutant HbS and wild type HbA are very similar as determined by X-ray diffraction. The two beta-6 residues in hemoglobin make up less than 1% of the total surface area in both proteins and a difference in pI of only 0.11. In the absence of conformational changes, a difference in the surface area and pI appears to account for the difference in the attractive interactions responsible for the self assembly of HbS into filaments. **Bottom:** The UVCD spectra for both HbA and HbS are characterized by a prominent minimum at 220 nm for alpha helix. The spectra are nearly identical despite the glu6val mutation which results in the self-assembly of HbS fibrils that produce sickling of red blood cells. The substitution of a hydrophobic valine (green) for a charged glutamate (red) at amino acid (β6) had no measurable effect on secondary, tertiary, or quaternary structure.

**Figure 3:**
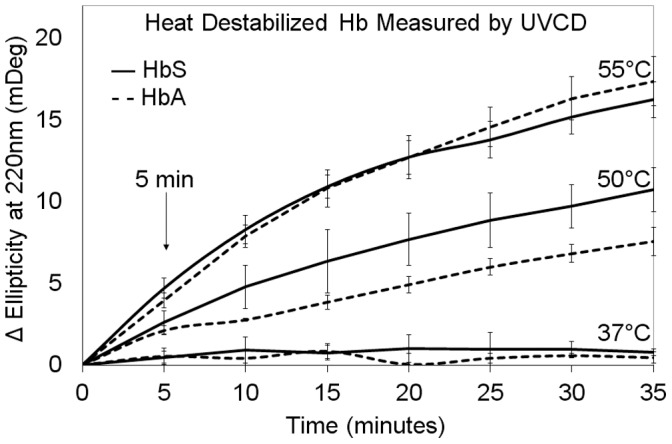
Destabilization of HbA or HbS with increasing temperature. Ellipticity at 220 nm was measured using UVCD every 5 minutes at 37, 50, or 55°C and the ΔEllipticity at 220 nm was recorded as the hemoglobin unfolded over time. The ΔEllipticity for HbA and HbS was similar when recording at 37°C and 55°C. At 50°C, the difference between HbS and HbA was statistically significant, but only after 10 minutes. At 5 minutes, thermal destabilization of HbS or HbA at 37°C, 50°C and 55°C was similar, as measured using ΔEllipticity. The 5 minute time point was chosen to evaluate the sensitivity of αB crystallin to HbA or HbS under thermal stress, prior to measurable unfolding.

200 µl of HbS, HbA, or αB crystallin at a concentration of 0.1 mg/ml in 150 mM PBS pH 7.0 were loaded into a 1 mm Quartz Spectrophotometer Cell (Starna Cells, Inc) and conformation was measured using a Jasco J-720 Circular Dichroism Spectrophotometer. The UVCD measurements were made at 37, 50, and 55°C. Readings at timepoint zero were taken immediately, and repeated at 5 minute intervals over a period of 35 minutes. The ellipticity (mdeg) was plotted on the Y-axis in figure 2. Ellipticity was observed between 205–270 nm with a bandwidth of 2 nm, a data pitch of 1 nm, and a speed of 200 nm/min. The change in ellipticity at 220 nm was a measure of the progressive destabilization of HbA or HbS at 37, 50, or 55°C. Destabilization was plotted in figure 3 as the change in ellipticity (ΔEllipticity) at 220 nm for HbA or HbS with time.

## Results

### Hemoglobin A and Hemoglobin S have Similar Secondary Structure and Thermal Stability

The effects of temperature on secondary structure were analyzed using far-UV circular dichroism (Figures 2 and 3). After 35 minutes at 37°C no change in ellipticity at 220 nm was measurable for either HbA or HbS (Fig. 3). During the 35 minutes of heating at 50°C the difference in ellipticity at 220 nm increased progressively to 7.6mDeg for HbA and 10.7mDeg for HbS. The effect on 3D conformation was greatest at 55°C. After 35 minutes the difference in ellipticity at 220 nm increased progressively to 17.4mDeg for HbA and 16.2mDeg for HbS (Fig. 3). The emphasis of this study was on the earliest significant changes in UVCD. After only 5 minutes at 50°C modest increases of 2.1mDeg for HbA and 2.6mDeg for HbS were observed. At 55°C, the unfolding was 3.7 and 3.9 mDeg for HbA and HbS respectively. The results indicated: (1) The progressive differences in structure increased with time, and the difference was greatest at 55°C >50°C >37°C, (2) No statistically significant differences between the unfolding of HbA and HbS at the same temperature were recorded using UVCD after only 5 minutes of heating. The 5 minute time point was selected for this experimental study of the sensitivity of αB crystallin to the earliest stages of thermal unfolding of HbA or HbS, when conformational changes and light scattering were minimal.

### αB Crystallin Prevented the Unfolding and Aggregation of HbA and HbS (Fig. 4)

**Figure 4:**
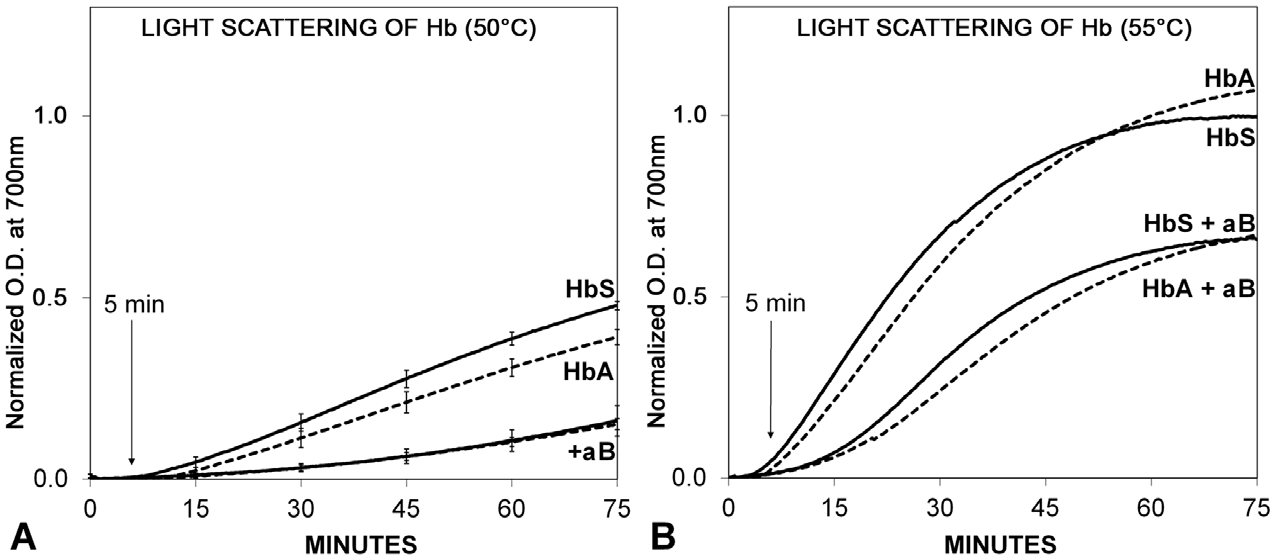
Thermal unfolding and aggregation of HbA or HbS in the presence or absence of αB crystallin. Aggregation was initiated by heating the sample to 50°C (A) or 55°C (B). **A.** In the absence of αB crystallin, light scattering was measured as optical density (O.D.) in milliabsorbance units (mAU) and normalized to the maximum observed at 55°C. The light scattering increased progressively with increasing protein unfolding and aggregation at 50°C. There was a measurable inhibition of aggregation of unfolding HbA or HbS in the presence of αB crystallin. **B.** At 55°C the protective activity of αB crystallin on thermal unfolding and aggregation of HbA and HbS was more obvious. The difference in light scattering for HbA and HbS in the presence or absence of αB crystallin was not statistically significant. No increase in light scattering was observed at 37°C (not shown) which was consistent with the absence of a change in the UVCD (Fig. 3). Aggregation and light scattering of unfolding HbA and HbS were minimal at 5 minutes which was consistent with the conformation measured using UVCD in Fig. 3. At all temperatures, αB crystallin inhibited the aggregation of unfolding HbA and HbS measured using light scattering.

Thermal aggregation assays of HbA and HbS were conducted to determine the effect of αB crystallin on light scattering resulting from the formation of insoluble aggregates of HbA and HbS (figure 4). As expected, the shape of the graphs of the change in optical density with time resembled those for a mechanism of nucleation and growth. The initial lag before the increase in optical density represented the earliest stages of destabilization prior to formation of insoluble, light scattering aggregates. After 75 minutes at 50°C in the absence of αB crystallin, light scattering at 700 nm increased gradually to 0.39 mAU (milli-Absorbance Units – see Methods) for HbA and to 0.47mAU for HbS (Fig. 4A). At 50°C in the presence of αB crystallin, the light scattering increased only to 0.15 for HbA and 0.16mAU for HbS. After incubation at 55°C in the absence of αB crystallin for 75 minutes, light scattering at 700 nm increased gradually to 1.07mAU for HbA and 1.00mAU for HbS (Fig. 4B). At 55°C, in the presence of αB crystallin the light scattering increased to 0.67mAU for HbA and to 0.66mAU for HbS. These results demonstrated that the protective effect of αB crystallin was similar against aggregation of HbA or HbS. Our study was most interested in the early stages of aggregation as measure by light scattering (O.D.). After only five minutes of heating, no increase in scattering was observed at 37°C (data not shown) or at 50°C. At 55°C a small increase in light scattering was observed for both HbA and HbS.

### Interactions between αB Crystallin and HbS or HbA at the Earliest Stages of Unfolding

**Figure 5:**
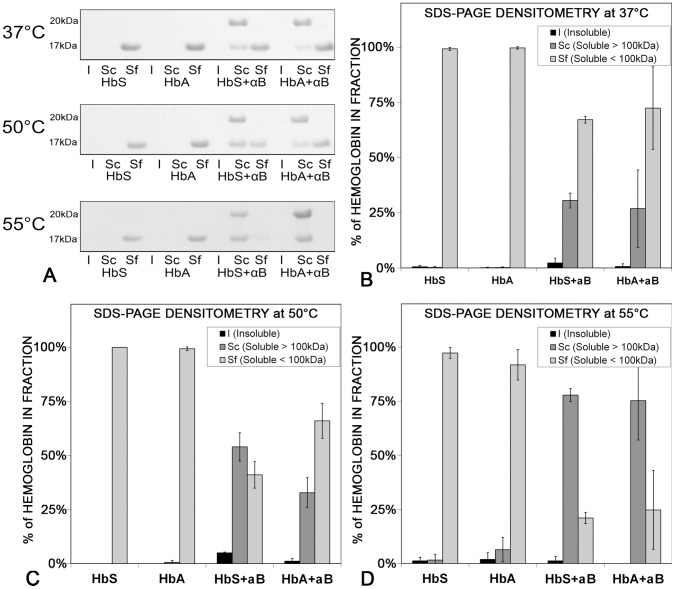
Interactions between HbA or HbS and αB crystallin increased after only five minutes of heating. A. SDS-PAGE of insoluble (I), soluble filtrate (Sf), and soluble concentrate (Sc). In the absence of αB crystallin (20 kD), the amounts of insoluble(I) aggregates were barely detectable after only 5 minutes at 37°C, 50°C or 55°C. Nearly all the HbA and HbS (17 kD) were in the soluble fraction (Sf). In the presence of αB crystallin, minimal interactions at 37°C resulted in a small amount of αB+HbA or αB+HbS in the soluble concentrate (Sc). At 55°C the interactions with αB crystallin were stronger and nearly all the HbA or HbS co-sedimented with αB in the soluble concentrate (Sc). **B–D.** Densitometry quantified the amounts of αB crystallin-bound and unbound HbA or HbS in the SDS-PAGE gels at each temperature. The X-axis shows the sample and the insoluble fraction: I (Insoluble), Sc (soluble-concentrate), or Sf (soluble-filtrate). The Y-axis is the measured percentage of protein in each fraction. **B.** At 37°C, the soluble unbound HbA and HbS were in the soluble filtrate (Sf) in the absence of αB crystallin. In the presence of αB crystallin, 27% of the HbA and 31% of the HbS was bound to αB crystallin in the soluble concentrate (Sc). The results demonstrated the weak interactions between αB crystallin and HbS or HbA even at 37°C for only 5 minutes. **C.** At 50°C, all destabilized HbA and HbS were in the soluble filtrate (Sf) in the absence of αB crystallin. In the presence of αB crystallin, the interactions between αB and destabilized HbS or HbA increased. The result was that 54% of the HbS-αB crystallin and 33% of the HbA-αB crystallin were in the soluble concentrate (Sc) at 50°C, an increase of 23% HbS and 6% HbA relative to 37°C after only 5 minutes. **D.** At 55°C, nearly all the unfolding HbA and HbS were in the soluble filtrate (Sf) in the absence of αB crystallin. In the presence of αB crystallin, the HbA or HbS bound to αB crystallin increased. Relative to 50°C and 37°C HbA-αB crystallin and HbS-αB crystallin were largely in the soluble concentrate (Sc) at 55°C The filtration assay confirmed an increase in interactions between αB crystallin and HbA or HbS with thermal destabilization after only 5 minutes when the differences in conformation were not significant. The interactions were stronger for HbS than HbA.

Sedimentation and 100 kDa spin-filtration analyses characterized the interactions between HbS or HbA and αB crystallin at the early stages of thermal destabilization (Fig. 5). After heating at 37°C, 50°C, or 55°C for only 5 minutes, samples were separated into insoluble (I), soluble concentrate (Sc), and soluble filtrate (Sf) fractions, analyzed using SDS-PAGE (Fig. 5A), and quantified using gel densitometry (Figs. 5B,C,D). At 37°C, 50°C, and 55°C in the absence of αB crystallin, the amount of insoluble aggregated HbA or HbS (17 kD) was so small as to be difficult to observe using SDS-PAGE of the insoluble (I) fraction. Nearly all HbA, HbS, and αB crystallin remained soluble in the soluble filtrate (Sf). The small amount of protein aggregation was consistent with the low levels of light scattering observed after five minutes of thermal unfolding in figure 4. After only 5 minutes at temperatures as high as 55°C, very weak molecular interactions between HbA or HbS accounted for the low level of light scattering and aggregation observed spectrophotometrically. In the absence of HbA or HbS at 37°C, 50°C, and 55°C, all of the αB crystallin was in the soluble concentrate (Sc)(data not shown) (26). After only 5 minutes at 37°C, αB crystallin was present in the soluble concentrate (Sc) co-sedimenting with HbA or HbS demonstrating an interaction between αB crystallin and HbA or HbS under normal, non-stress conditions.

Quantitatively, 2% of the HbS was measured in the insoluble (I) fraction after 5 minutes at 37°C in the presence of αB crystallin, while 31% of the HbS was measured in the soluble concentrate (Sc), and 67% was measured in the soluble filtrate (Sf) (Fig. 5B). Similarly, 1% of the HbA was measured in the insoluble (I) fraction, 27% was measured in the soluble concentrate (Sc), and 72% was measured in the soluble filtrate (Sf) at 37°C (Fig. 5B). Less HbA (27%) than HbS (31%) co-sedimented with αB crystallin at 37°C. These results were a quantitative measure of the differences in the interactions between αB crystallin and either HbA or HbS at 37°C in the absence of statistically significant differences in UVCD or light scattering.

After 5 minutes at 50°C, the interactions between αB crystallin and destabilized HbA or HbS, increased as measured by the co-sedimenting protein in the soluble concentrate(Sc) and insoluble (I) fraction (Fig. 5C). The amount of HbS co-sedimenting with αB crystallin at 37°C and 50°C increased from 31% to 54% in the soluble concentrate (Sc) and from 2% to 5% in the insoluble (I) fraction. There was a corresponding decrease from 67% to 41% of soluble HbS in the soluble filtrate (Sf) at 50°C. For HbA at 37°C and 50°C, 1% of the HbA-αB crystallin co-sedimented in the insoluble (I) fraction, while the amount of destabilized HbA co-sedimenting with αB crystallin in the soluble concentrate (Sc) increased from 27% to 33%. There was a corresponding decrease of soluble HbA from 72% to 66% in the soluble filtrate (Sf). While the amount of HbS-αB crystallin was approximately 6% greater than HbA-αB crystallin protein after 5 minutes at 37°C, at 50°C, the amount HbS-αB crystallin was more than 20% greater than HbA-αB crystallin after 5 minutes, even though UVCD determined no measureable differences in the conformation of HbS or HbA. The results in figure 5 illustrate that the interactions between αB crystallin were stronger for destabilized HbS than HbA. Most importantly for this study of early sensitivity, interactions with αB crystallin were stronger for HbS than HbA at both 37°C and 50°C after only 5 minutes. The results of the sedimentation assay were consistent with the hypothesis that αB crystallin is more sensitive to the mutant, self-assembling HbS than the stable, soluble HbA, even in the absence of measureable changes in conformation or increased light scattering at the earliest stages of thermal destabilization.

## Discussion

The results indicate that human αB crystallin is extremely sensitive to protein destabilization prior to unfolding of normal (HbA) and sickle cell (HbS) hemoglobin. Interactions between human αB crystallin and HbA or HbS were observed before measureable changes in conformation or increased light scattering due to thermal stress were significant. The interactions increased with increasing temperature. On the basis of these results, the initiation of effective protection against protein unfolding and aggregation under conditions of cellular stress can be expected to occur before nucleation and growth results in the irreversible formation of insoluble fibrils and/or protein aggregates and well before the disruption of normal cellular function.

The results confirmed that differences in the surfaces of HbS and HbA resulting from the Glu6Val substitution were recognized by αB crystallin and could account for the greater sensitivity to destabilized HbS than HbA, in the absence of measurable conformational changes. Previous X-ray diffraction and biochemical studies found that HbS differed from HbA in hydrophobic surface area by approximately 1% as a result of the Glu6Val substitution in the mutant HbS. The difference in isoelectric point was only 0.11 [26] and pI is known to be a measure of attractive forces in protein interactions with αB crystallin [27]. While differences as small as 0.11 in the pI and 1% of the hydrophobic surface area are not expected to be functionally important, they appear to account for the differences in the initial interaction of HbA and HbS with αB crystallin, which is a very sensitive detector of molecular destabilization in vitro. Future experiments will characterize the intracellular sensitivity of αB crystallin in vivo to destabilizing changes during protein unfolding and aggregation using non-invasive microscopic methods.

While UVCD is a conventional measure of 3D conformational changes in proteins, increased light scattering is a common measure of increased molecular dimensions resulting from attractive interactions between aggregating proteins. Light scattering is a simple analytical technique for evaluation of the protective activity of αB crystallin against protein unfolding and self assembly to aggregates. In contrast, the sedimentation assay (fig. 1) separated the interacting proteins by size and determined the amount of each constituent protein using SDS-PAGE. Taken together, the three assays demonstrated that variations in interactions resulting from differences in hydrophobic surface area or pI were sufficient for recognition by human αB crystallin at the earliest stages of protein destabilization prior to unfolding measured using UVCD or light scattering.

The increase in αB crystallin bound HbA or HbS with increased temperature was measureable using SDS-PAGE of the centrifuged samples after only five minutes (fig. 5), even though statistically significant changes in structural conformation were not measureable using UVCD or light scattering. After only five minutes, the interactions between αB crystallin and HbA or HbS increased the amount of co-sedimenting protein more than two-fold with an increase in temperature from 37°C to 50°C. The increase in destabilized HbS or HbA bound αB crystallin corresponded to a change of only 2.2 mDeg measured using UVCD and 0.0 in normalized light scattering. Even though the structural difference between destabilized wild-type HbA and mutant HbS measured using UVCD or light scattering was insignificant, it was detected by αB crystallin. More destabilized HbS than HbA interacted with αB crystallin which is consistent with αB crystallin as a molecular sensor for surface instabilities leading to protein aggregation and/or amyloid formation in disorders of aging including neurodegeneration and cataract. The results suggested that little or no unfolding was required for recognition of differences in destabilized self-assembling proteins during the initiation of a protective response by αB crystallin.

### In Summary

The results of the current study were consistent with the hypothesis that the stress response protein, human αB crystallin, is extremely sensitive to surface modifications at the earliest stages of molecular destabilization leading to protein self assembly. The experiments demonstrated the remarkable capability of αB crystallin to recognize unmeasurable differences between normal and abnormal proteins under conditions of thermal stress prior to aggregate formation. This study confirmed experimentally that aB crystallin can detect destabilized proteins before conformational changes can be measured in vitro using conventional spectroscopic techniques.
